# The Effect of Usage of Antiasthmatic Inhalers on Color Stability and Surface Roughness of Dental Restorative Materials: An *In* *Vitro* Study

**DOI:** 10.1055/s-0043-1771450

**Published:** 2023-08-10

**Authors:** Priya Subramaniam, Devikripa Bhat, Megha Gupta, Shabnam Gulzar, Altaf H. Shah

**Affiliations:** 1 Department of Pedodontics and Preventive Dentistry, The Oxford Dental College and Hospital, Bangalore, Karnataka, India; 2 Department of Pedodontics and Preventive Dentistry, Vyas Dental College and Hospital, Jodhpur, Rajasthan, India; 3 Pedodontics and Preventive Dentistry, District Hospital Pulwama, Pulwama, Kashmir, India; 4 Special Care Dentistry Clinics, University Dental Hospital, King Saud University Medical City, Riyadh, Saudi Arabia

**Keywords:** asthma, antiasthmatic agents, composite resins, dental materials, dental restoration

## Abstract

**Objectives** Increased demand for esthetics by parents and children has resulted in the use of tooth-colored restorative materials. Children with chronic respiratory conditions like asthma use inhalers which have shown to affect the surface of restorative materials. Hence, the aim of the present study was to evaluate the effect of antiasthmatic inhalers on color stability and surface roughness of three restorative materials.

**Materials and Methods** Forty samples each of three dental restorative materials: group A: glass ionomer cement (GIC), group B: alkasite restorative material, and group C: composite resins were prepared. Each group was further divided into two subgroups of 20 samples each according to the inhaler used. All the specimens were polished using polishing discs and stored in artificial saliva in order to simulate the oral environment. The baseline color value and surface roughness of all the samples were measured using a spectrophotometer and a profilometer, respectively. Group 1 and group 2 were exposed to 0.31 mg of salbutamol sulfate and 20 mg formoterol fumarate in combination with budesonide, respectively, for every 12 hours, for a period of 15 days following which the samples were evaluated for color changes and surface roughness.

**Statistical Analysis** The data obtained was subjected to statistical analysis and level of significance was set at *p* < 0.05.

**Results** Exposure to both the inhalers caused a change in color and surface roughness in all three restorative materials. There was a significant change in the color of GIC and composite resin (ΔE > 3.3), following exposure to both the inhalers ( *p* < 0.05). The change in color of alkasite restorative material was not significant. A significant increase in the surface roughness of composite resin from 0.56 ± 0.14 to 0.67 ± 0.19 was seen following 15 days' exposure to formoterol in combination with budesonide inhaler ( *p* < 0.05).

**Conclusion** Following exposure, both the inhalers had an equal effect on color and surface roughness of all three restorative materials. Alkasite restorative material showed greater resistance to change in color and surface roughness when exposed to antiasthmatic inhalers, compared to GIC and composite resin. Thus, children who use inhalers and nebulizers should be advised to implement more precautionary oral hygiene measures and periodic dental visits.

## Introduction

Increased demand for esthetics by parents and children has resulted in the use of tooth-colored restorative materials in pediatric dentistry. An ideal esthetic restorative material must mimic the natural tooth in color, translucency, surface texture, and show color stability over a prolonged period. Glass ionomer cement (GIC) and composite resins are commonly used in restoration of primary teeth. 1

The requirement for superior esthetic restorations has led to the development of newer materials with improved properties. Recently, an innovative metal-free esthetic alkasite material which is a subgroup of composites has been introduced with improved flexural strength, compressive strength, sorption, and solubility. It has superior esthetics when compared to GIC. Fluoride and hydroxyl ion-releasing properties makes it cariostatic. 2 3

Smart monochromatic composite is also promising as it decreases the requirement of a range of composite shades, curtails the waste of unconsumed composite shades, lessens chairside interval, and avoids the shade selection process. These composites obtain the color of the adjacent tooth structure in which it is placed and hence are superior than conventional composites. 4

The longevity of a restorative material is dependent on its color stability and surface roughness. 5 6 7 8 Since the oral cavity has a dynamic environment, it is a challenge to maintain the color stability of materials. The color of restorative materials depends on its surface spectral reflectance, which is a sensitive function of its roughness. 9 Hence, surface roughness of a material is of considerable importance as it influences the optical properties of the material. 10 11 The color of restorative materials can be influenced by extrinsic factors such as foods, beverages, medications, and oral hygiene practices. The inherent properties, composition, and polymerization of restorative materials are the intrinsic causes for discoloration. 12 13

Asthma is a complex chronic inflammatory disease of the lower airways and is characterized by variable airflow obstruction and airway hyperresponsiveness. 14 15 The prevalence of this condition in children has been increasing from 2 to 3% to 8 to 19% in both genders in different parts of the world. 16 17 These children require medications on a daily basis, which may be in the form of syrups, capsules, metered-dose inhalers, or dry powder inhalers. 18 The composition of inhalers includes active medicament, acid, and probably alcohol. The plume temperature, velocity, pH, and ingredients of inhalers could have an effect on the teeth, mucosa, and dental restorations. 14

Numerous studies have reported on the effect of foods, beverages, and liquid medications on restorative materials. 8 19 20 21 22 Surface roughness and color properties are related to each other for dental materials because restorations with high surface roughness are more susceptible to staining. Increased surface roughness causes plaque accumulation and discoloration of the material. 23 Additionally, the surface texture affects the color of the restoration, as a smooth surface reflects a greater amount of light than a rough surface. 24 However, there are very few studies that have assessed the effects of antiasthmatic inhalers on the dental restorative materials. Ayaz et al 14 concluded that inhaler treatment with salbutamol sulfate significantly increased the surface roughness and color change of GIC and composite resin materials, while the surface roughness and color of feldspathic porcelain was not changed after inhaler treatment, that is, the intrinsic properties and finishing procedures of restorative materials affected the surface roughness characteristic. Moreover, porcelain materials do not change very much during their life in the oral cavity, but composite-based materials suffer degradation due to mechanical and/or chemical interactions with the oral environment. 25 Additionally, dental materials composed of composite resins may absorb water and chemicals from the oral environment, which may affect the surface roughness. This discoloration of the restorative materials in patients using salbutamol inhaler may possibly be due to the active ingredient of the inhaler nebule, which contains a (C _13_ H _21_ NO _3_ ) 2·H _2_ SO _4_ sulfate group. The ingredients of this drug may affect the surfaces of the dental materials by forming a pellicle matrix that provides an acidic environment, thus promoting demineralization and increasing surface roughness and discoloration. 14 Therefore, the aim of this study was to investigate the effect of antiasthmatic inhalers on the color stability and surface roughness of restorative materials, namely, GIC, alkasite restorative material, and composite resin.

## Materials and Methods

This *in vitro* study was carried out at the Department of Pedodontics and Preventive Dentistry and ethical clearance was obtained from the Institutional Ethics Review Board (Ref No. 179/ECAL/2019-20).

### Estimation of Sample Size

The sample size was estimated using NMaster 2.0 software. Considering the mean and standard deviation of population and sample for two-tailed hypothesis, power of the study 90%, and marginal error at 1%, the required sample size came up to 13 per group which was rounded off to 20 per group:

where,
*σ* : standard deviation
*x* : sample mean
*µ* : population mean
*δ* : effect size
*α* : significance level
1– *β* : power

A total of 120 standardized molds, each measuring 10 mm in diameter and 3 mm in thickness, were prepared using light body impression putty (Zhermack Hydrorise Putty and Light Body Impression Material, Polesine, Italy). These molds were further divided equally into three groups ( *n* = 40) for preparation of the sample discs of three dental restorative materials: group A: GIC (GC Gold Label 2, GC Asia Dental Pte Ltd, Loyang Way, Singapore), group B: alkasite cement (Cention N, Ivovlar Vivadent AG, Benderer Str, Liechtenstein), and group C: composite resin (Filtek Z350 XT, 3M ESPE, St. Paul, Minnesota, United States). The curing and setting of the samples was done according to the manufacturer's instructions.

In groups B and C, alkasite restorative material and composite resin, respectively, were placed in the molds in increments cured for 20 and 40 seconds, respectively, using Light emitting diode (LED) of 430 to 490 nm intensity (LEDtion, Ivoclar Vivadent AG); while in group A, GIC was mixed according to the manufacturer's instructions and covered with a Mylar strip to obtain a smooth surface. All the samples were then polished using polishing discs (SHOFU Dental ASIA-Pacific Pte. Ltd., Science Park Road, Singapore).

The samples in each group were divided into two subgroups of 20 samples each; group 1: levosalbutamol inhaler (Aerozest 250MD inhaler, Macleods Pharmaceuticals Ltd, Bangalore, Karnataka, India) and group 2: formoterol fumarate in combination with budesonide inhaler (Budamate 200MD inhaler, Lupin Pharmaceuticals, Mumbai, Maharashtra, India). Following the preparation of artificial saliva, the samples were incubated in artificial saliva for 24 hours at 37°C in order to simulate the oral environment.

The baseline color measurements and surface roughness of all the samples were measured using spectrophotometry (Data color 650TM, Data color Technology Co. Ltd., Lawrenceville, New Jersey, United States) and profilometry (Mitutoyo, Surftest SJ-210 Series, Kanagawa, Japan), respectively. Group 1 was exposed to 0.31 mg of levosalbutamol and group 2 was exposed to 20 mg formoterol fumarate in combination with budesonide every 12 hours for 15 days, after which the samples were again evaluated for changes in color and surface roughness.

Color changes from baseline were examined using spectrophotometry according to the Commission International de I Eclairege L* a* b* (CIELAB) color space system using the formula:1-5 δ E (L*a*b*) = [(δ L*) ^2^ + (δ a*) ^2^ + (δ b*) ^2^ ] ^½^ , where, δ E is the color difference of the samples, δ L* is the difference between L* values, δ a* is the difference between a* values, and δ b* is the difference between b* values. Here, L* represents brightness or lightness (value) and a* and b* represent red/green and yellow/blue, respectively. Three Ra values of the samples were measured at the center of each sample with profilometry using 0.4-gf load for 5s and the arithmetic mean was calculated.

Data obtained were subjected to two-way analysis of variance and Tukey's post hoc analysis to compare the mean difference between three different restorative materials under two different parameters (color stability and surface roughness). A paired sample *t* -test was performed to measure the mean difference of surface roughness and color stability of the restorative materials before and after exposure to the drug. The significance difference was set to *p* < 0.05.

## Results

Table 1 describes the mean color values of the restorative materials at baseline and after exposure to the inhalers. The color values after 15 days of exposure to both the inhalers show a decrease in all the three restorative materials, with a significant decrease in groups A and C following 15 days of exposure to both the inhalers ( *p* = 0.001). In group B, that is, alkasite restorative material, the change in mean color value was not significant.

**Table 1:** Comparison of mean color value of restorative materials following exposure to inhalers

Restorative material	Inhaler	At baseline Mean ± SD	After 15 days Mean ± SD	*p* -Value
Glass ionomer cement (GIC) (group A)	Salbutamol sulfate inhaler	75.43 ± 1.36	72.54 ± 1.39	0.001 a
	Formoterol fumarate + Budesonide inhaler	75.79 ± 1.39	72.89 ± 1.68	0.001 a
Alkasite restorative material (group B)	Salbutamol sulfate inhaler	61.9 ± 3.0	61.8 ± 2.12	0.852
	Formoterol fumarate + Budesonide inhaler	61.9 ± 3.0	60.88 ± 2.27	0.948
Composite resin (group C)	Salbutamol sulfate inhaler	68.3 ± 0.99	66.76 ± 2.35	0.007 a
	Formoterol fumarate + Budesonide inhaler	68.22 ± 0.39	66.49 ± 2.62	0.009 a

Abbreviation: SD, standard deviation.

a *p* < 0.05 is significant.

Table 2 compares the mean surface roughness of the restorative materials following exposure to inhalers. The mean surface roughness after the use of group 1 (salbutamol sulfate) inhaler decreased in all the restorative materials, but it was not statistically significant. The mean surface roughness of group C, that is, composite resins, significantly increased following exposure to group 2 (formoterol fumarate + budesonide) inhaler ( *p* = 0.01). Table 3 depicts the intergroup comparisons of the mean color value and surface roughness. At baseline and following exposure to both the inhalers, there was a significant difference in mean color among the three restorative materials ( *p* = 0.001). A significant difference in surface roughness was seen between the restorative materials following 15 days' exposure to both the inhalers ( *p* = 0.03).

**Table 2:** Comparison of mean surface roughness of restorative materials following exposure to inhalers

Restorative material	Inhaler	At baseline Mean ± SD	After 15 days Mean ± SD	*p* -Value
Glass ionomer cement	Salbutamol sulfate inhaler	0.96 ± 0.57	0.81 ± 0.34	0.282
	Formoterol fumarate + Budesonide inhaler	0.84 ± 0.38	0.91 ± 0.59	0.313
Alkasite restorative material	Salbutamol sulfate inhaler	0.96 ± 0.57	0.81 ± 0.34	0.282
	Formoterol fumarate + Budesonide inhaler	0.84 ± 0.38	0.91 ± 0.59	0.313
Composite resin	Salbutamol sulfate inhaler	0.66 ± 0.28	0.57 ± 0.19	0.136
	Formoterol fumarate + Budesonide inhaler	0.56 ± 0.14	0.67 ± 0.19	0.01 a

Abbreviation: SD, standard deviation.

a *p* < 0.05 is significant.

**Table 3:** Intergroup comparison of mean color value and surface roughness of restorative materials following exposure to inhalers

Inhalers	Groups (restorative materials)	Time			
		Mean color value		Surface roughness	
		Baseline *p* -value	After 15 days *p* -value	Baseline *p* -value	After 15 days *p* -value
Group 1 (salbutamol sulfate)	Group A (glass ionomer cement)	0.001 a	0.001 a	0.11	0.03 a
	Group B (alkasite restorative material)				
	Group C (composite resin)				
Group 2 (formoterol fumarate + budesonide)	Group A (glass ionomer cement)	0.001 a	0.001 a	0.09	0.03 a
	Group B (alkasite restorative material)				
	Group C (composite resin)				

a *p* < 0.05 is significant.

## Discussion

The present study showed that the inhalation of salbutamol sulfate and formoterol fumarate inhalers significantly affected the properties of GIC and composite resins, but it did not produce any significant changes in alkasite restorative materials.

The surface properties of restorative material play a major role in the long clinical life of restoration. 26 Color and surface roughness of restorative materials are properties dependent on each other. 27 28 Previous studies have reported that an increase in the surface roughness allows stain penetration. 14 17 A recent study concluded that the structure and composition of composites and compomer materials greatly affect the wear resistance. This comprises of the matrix characteristics, type of filler, and filler-particle size. 26 Majority of the children with asthma, use metered-dose inhalers over prolonged periods. 14 There have been several studies conducted on the effect of foods and beverages on esthetics of restorative materials. 21 29 The effect of medicated syrups on color stability and surface roughness have been evaluated. 19 22 However, similar studies on effect of antiasthmatic inhalers on restorative materials is lacking. 14

GIC possess cariostatic properties related to their sustained fluoride release and long-term adhesion to tooth structure. Composite restorations are considered to be highly esthetic and mimic the natural tooth structure. 30 Alkasites are a subgroup of composite resins that have been recently introduced and are considered to be similar to composite resins in esthetics and handling characteristics. The high translucency allows alkasites to blend in naturally with the surrounding tooth structure, while covering discolored dentin at the same time.

In the present study, samples of GIC, alkasite restorative material, and composite resin were prepared in molds made from light body impression putty, because of its high accuracy, good dimensional stability, and high tear strength. 31 32 33 34 Finishing and polishing of restorations enhance their esthetics and longevity and reduces the probability of stain penetration. Hence, all the materials were polished. Samples of the restorative materials were only placed in artificial saliva and incubated at 37°C. 29 Artificial saliva is a colorless medium and has been reported to have no effect on the color of restorative materials. 20

The CIE Lab system is commonly used by dental researchers to examine materials with regard to their color as it provides both a color difference formula and correlates for common perceptual descriptors of color. 35 This color system can transform spectrophotometer data to an approximately uniform color space. 13 In the present study, CIE Lab color system was used to estimate color stability and a white background was used as an illuminant against which the color difference (ΔE) was tested. This ΔE value represents relative color values of restorative materials prior to and following an intervention. 36 In the current study, a digital profilometer was used for the measurement of change in the surface roughness of restorative samples. The average roughness value (Ra) was used to describe surface roughness. 8 27 Profilometry is a direct technique that provides a two-dimensional measurement with advantages of acceptance, surface independence, and good resolution when compared to atomic force microscopy and rugosimetry. 8

The reflectance of a material refers to the ratio of the total amount of light that is reflected from its surface compared to the total amount originally incident upon it. This is dependent on its surface roughness and its optical properties. 9 10 The three materials used in this study differed in their inherent composition, optical properties, water sorption, and solubility. 37 Therefore, at baseline itself a significant difference in color was seen between the materials against a white background. However, the difference in their surface roughness was comparable.

The two inhalers used in this study differed in their composition, fine particle size plume velocity, and their pH ranged between 3 and 4. During inhaler use, the sulfuric acid in salbutamol sulfate inhaler and citric acid in formoterol fumarate + budesonide inhaler produces an acidic environment around each material. The sulfuric acid present in the salbutamol sulfate inhaler has a lower pKa value which gets reduced to sulfur dioxide due to the presence of alcohol, thus making it less erosive. 38

The presence of H ^+^ ions causes leaching of Ca ^+2^ or Al ^+3^ ion, from GIC. As the metal cations in the matrix decrease, the dissolution around the glass particles increases. The pits thereby formed by the dislocation of glass particles and the ledges formed by the undissolved glass particles result in the increased surface roughness. 20 In the present study, Ra vales were of 0.81 and 0.91, following exposure to salbutamol sulfate and formoterol fumarate + budesonide, respectively. Further, long time exposure of GIC can form cracks in the material surface due to its water sorption property. There is an alteration in surface texture of the material which could be the reason for significant change in its color stability. GIC showed the highest ΔE values following exposure to both inhalers, 3.5 and 3.79, respectively These findings are similar to that of Ayaz et al. 14

Alkasite restorative material contains an alkaline filler, calcium fluorosilicate, which releases hydroxyl ions that could have probably neutralized the acidic environment caused by the inhalers. 39 40 Therefore the material showed least change in surface roughness with Ra values of 0.69 and 0.59 on exposure to salbutamol sulfate and formoterol fumarate + budesonide, respectively. The liquid component of alkasite restorative material contains urethane dimethacrylate (UDMA), which is a hydrophobic, high-viscosity cross-linker having low tendency to discolor. With UDMA, a rigid network is formed resulting in lower water sorption and higher release of unreacted monomer. 41 42 This could be the probable reason for a negligible color change of ΔE.

In comparison to alkasites, the composite resin used in this study contains bisphenol A-glycidyl methacrylate and triethylene glycol dimethacrylate, which are hydrophilic in nature, leading to increased water sorption. The acidic content present in both the inhalers resulted in the surface degradation of the material. There is associated softening of the polymer matrix and displacement of organic filler in the composite resin. 14 36 43 However the surface roughness was significant only on exposure to formoterol fumarate + budesonide. It was probably due to the lower plume velocity of the formoterol fumarate + budesonide inhaler, resulting in its slower and less forceful drug expulsion over the softened polymer matrix. The fillers of the composites are described as nanosized clusters formed by aggregated zirconia/silica nanoparticles, which may have porosities. 44 45 There is a high probability of aerosol droplets getting absorbed into these porosities causing discoloration of the composite resin with ΔE of 3.4.

Dental restorations are exposed to masticatory stresses, saliva, biofilm, food intake, and oral hygiene practices. Variations in pH and temperature of the oral cavity are also factors to be considered. Subtle changes in the color and surface roughness of restorations are not visible to the naked eye and cannot be evaluated clinically. However, simulation of the complex oral environment is difficult to achieve for *in vitro* studies on surface texture. 46 Since there is a paucity of literature on the effects of antiasthmatic inhalers on dental restorative materials, it limited the comparison of our findings with similar studies. Further, investigations using atomic force microscopy and three-dimensional images can be carried out to provide quantitative data of surface characteristics.

## Conclusion

Although GIC is anticariogenic and bond chemically to the tooth structure, its color stability is compromised. Composite resins are considered to be highly esthetic and are available in several shades, but its inherent composition and properties could be a deterrent for color stability. Alkasite is a less technique sensitive, biocompatible, and fluoride-releasing material which showed better color stability. Alkasite restorative material appears to be a promising alternative for esthetic restorations in children using antiasthmatic inhalers.
